# Supplementary material for: The Bartonella autotransporter BafA activates the host VEGF pathway to drive angiogenesis
Source: Nat Commun. 2020 Jul 16;11:3571. doi: 10.1038/s41467-020-17391-2 (PMC7366657; doi:10.1038/s41467-020-17391-2)
Supplement: Supplementary file 1 — Supplementary Information [file 41467_2020_17391_MOESM1_ESM.pdf]

Supplementary Information

The *Bartonella* autotransporter BafA activates the host VEGF pathway to drive angiogenesis

Tsukamoto et al.

Supplementary Figures 1–10

Supplementary Tables 1–4

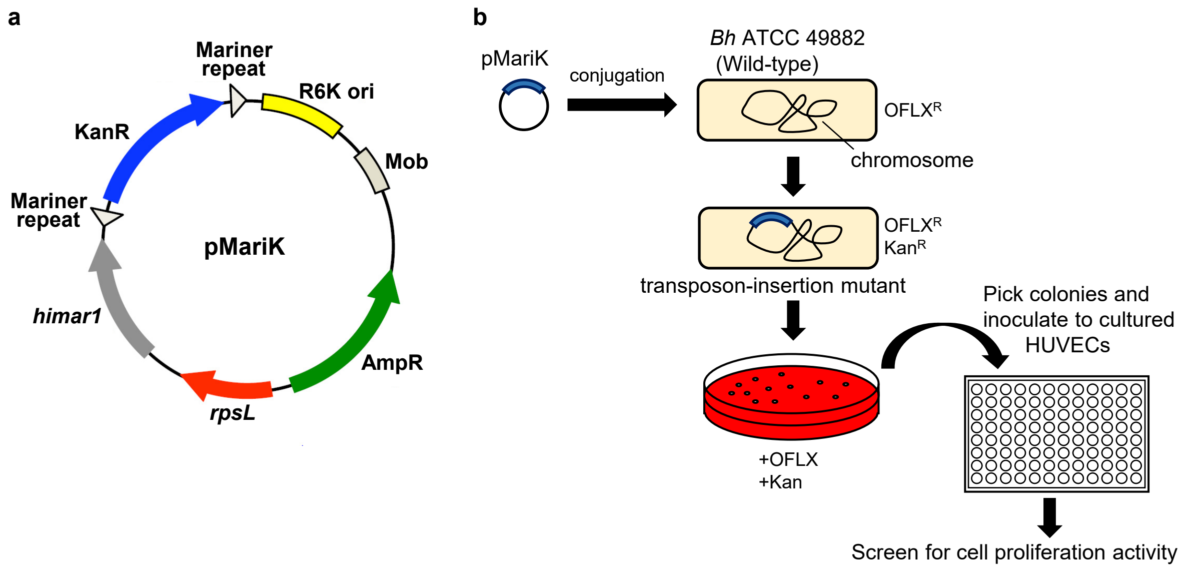

Supplementary Figure 1. Generation of *Bh* transposon-insertion library. a, Plasmid map of a *mariner*-based transposon vector pMariK. The plasmid contains genes for antibiotic resistance (ampicillin, AmpR; kanamycin, KanR), origin of replication (R6K ori), origin of transfer (oriT), oriT-recognising protein (*traJ*), transposase (*himar1*), 30S ribosomal protein S12 (*rpsL*) and mariner inverse repeats. **b**, Schematic diagram from generation of transposon-integrated mutants to functional screening.

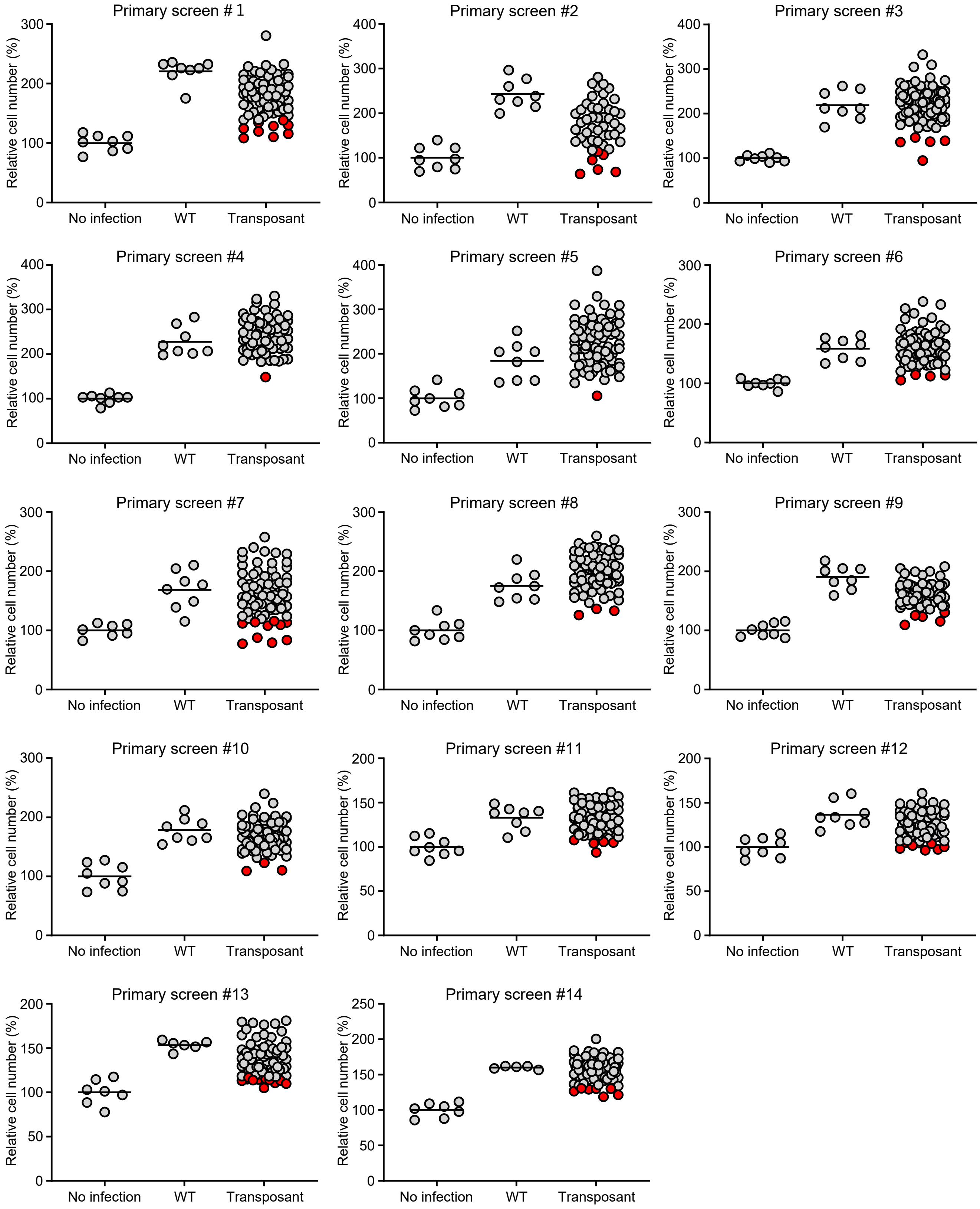

Supplementary Figure 2. Primary screen for *Bh* transposants deficient in cell proliferation ability. HUVECs were infected with *Bh*-WT or transposants in 96-well plates, and the cells were counted using 6–8 wells for WT or one well for each transposant after 3 days of culture. The relative cell numbers are shown as circles, and mean values as lines (the mean of no infection controls = 100%, n = 6–8 biological replicates). From 1,090 transposants, 79 transposants (red) with reduced cell proliferation activity were selected over 14 assays. Source data are provided as a Source Data file.

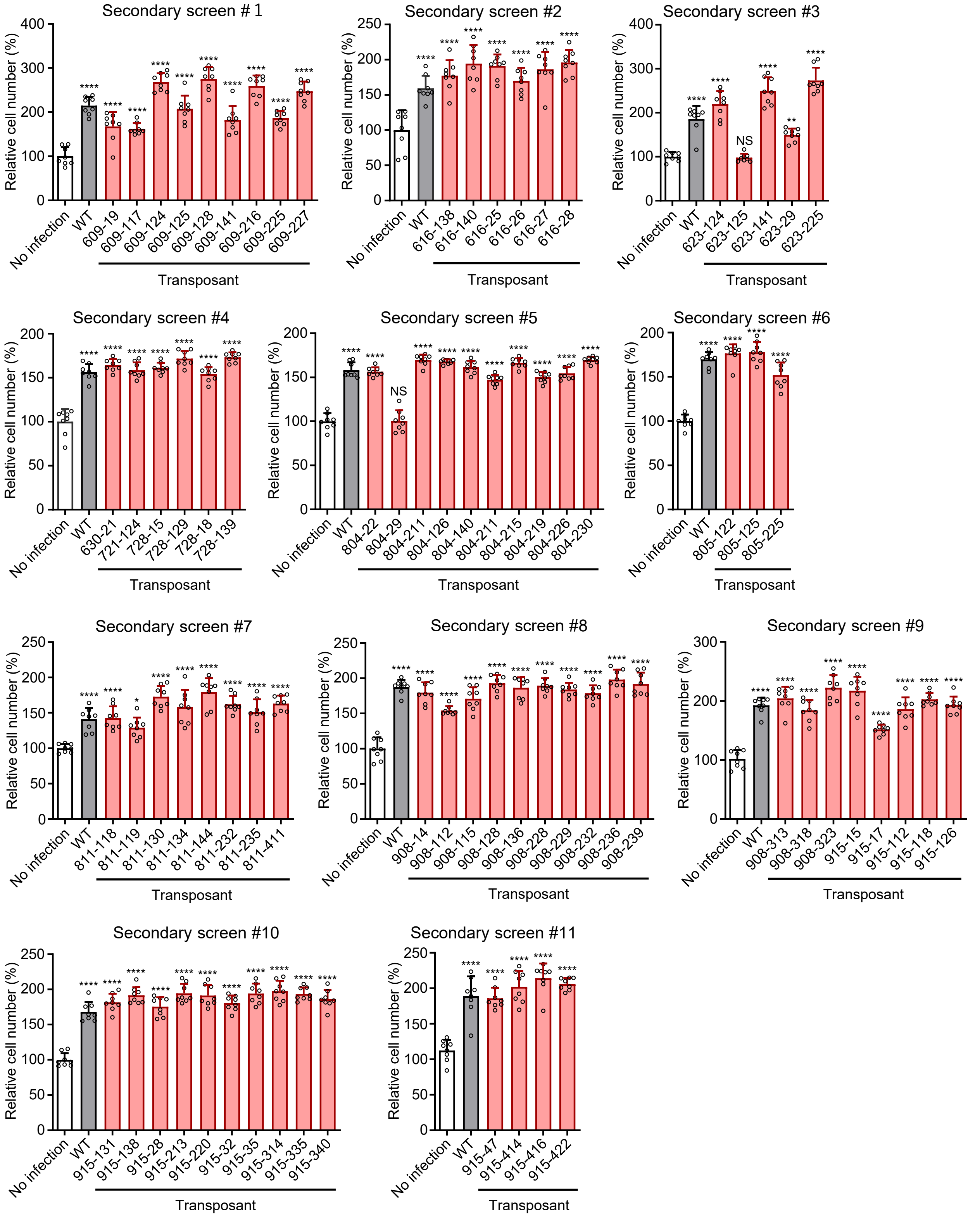

Supplementary Figure 3. Secondary screen for *Bh* transposants deficient in cell proliferation ability. HUVECs were infected with *Bh*-WT or transposants at MOI of 300 in a 96-well plate, and the cells were counted using 8 wells for each transposant after 3 days of culture. From 79 transposants selected by the primary screen, 2 transposants (623-125 in screen #3 and 804-29 in screen #5) were obtained that lacked cell proliferation ability in 11 assays. Bars are coloured based on the infecting strains: white for no infection, grey for WT and red for transposants. Data are mean ± s.d. (n = 8 biological replicates). Statistical significance was determined using one-way ANOVA with Dunnett's multiple comparisons test. *P < 0.05; **P < 0.01; ****P < 0.0001; NS, not significant, compared with no infection control. Source data are provided as a Source Data file.

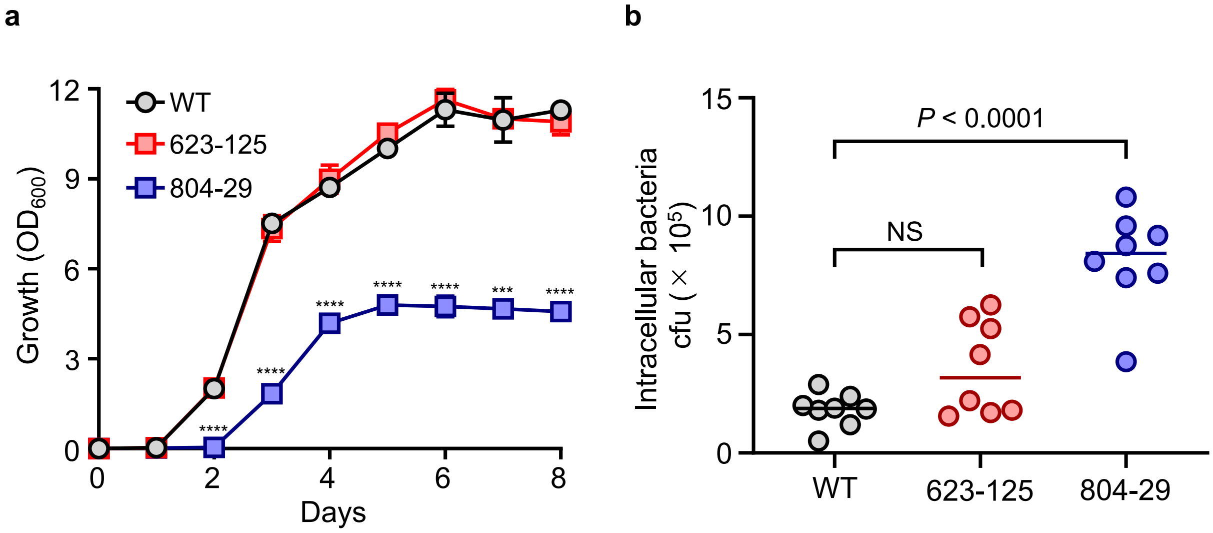

Supplementary Figure 4. Bacterial growth and invasion of *Bh* into HUVEC. a, Course of bacterial growth on Columbia agar plates. *Bh*-WT, transposants 623-125 and 804-29 were cultured on the agar plates. After the indicated days, bacteria were harvested and resuspended in PBS followed by OD_{600} measurement. Data are mean ± s.d. (n = 3 biological replicates). Statistical significance was determined using two-tailed Student's t test. ***P < 0.001; ****P < 0.0001, compared with WT. **b,** Number of intracellular bacteria after 24 h of infection. After the infection, extracellular bacteria were killed by gentamicin treatment for 2 h, followed by washing steps and lysis of the cells. The cfus in cell lysate were determined by plating on Columbia agar. The values are shown as circles, and mean values as lines (n = 8 biological replicates). Each group is coloured based on the strains: grey for WT, red for 623-125 and blue for 804-29. Statistical significance was determined using one-way ANOVA with Dunnett's multiple comparisons test. Source data are provided as a Source Data file.

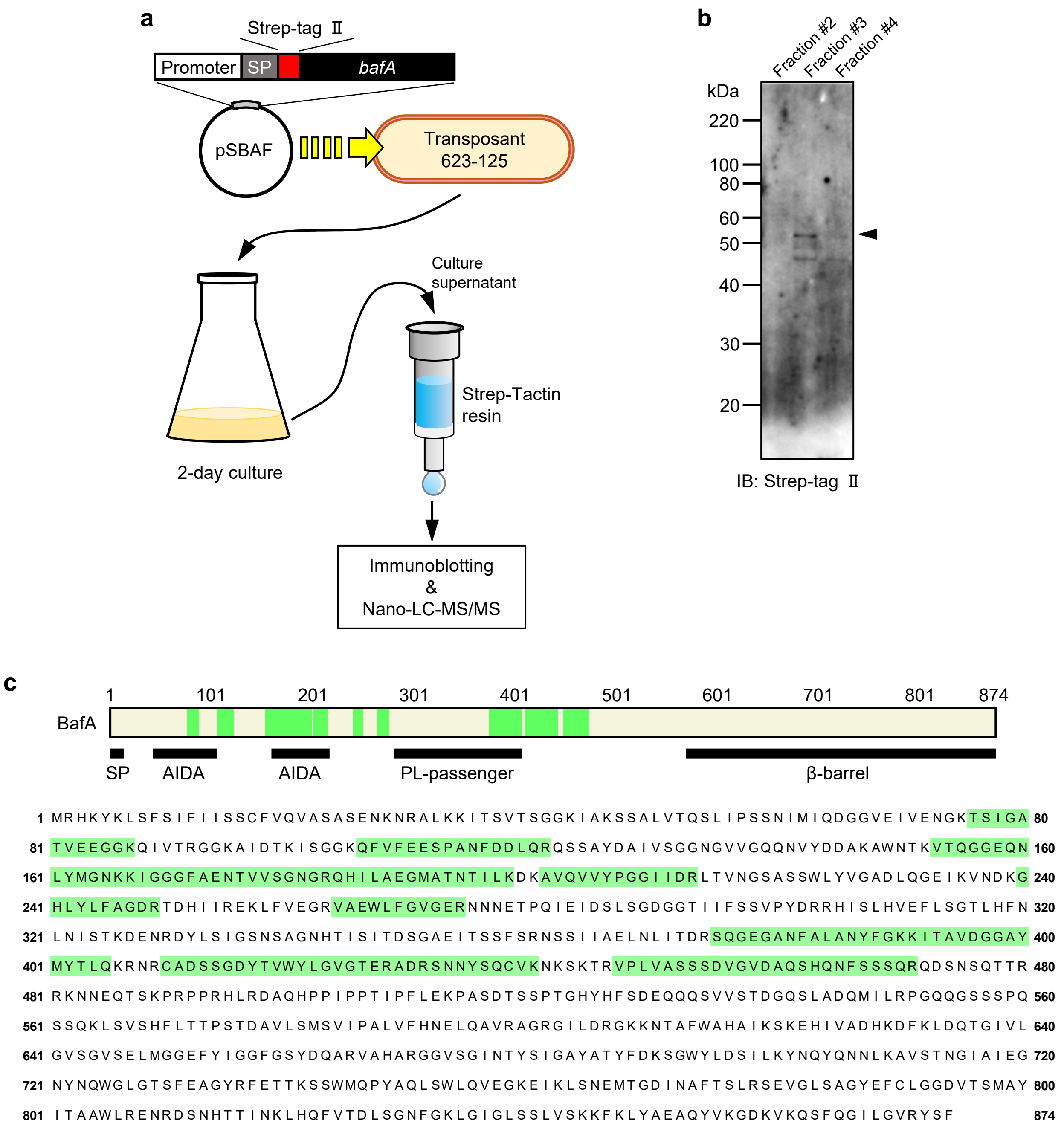

Supplementary Figure 5. The passenger domain of BafA is secreted by *Bh*. a, Schematic diagram from generation of a Strep-tagged BafA-expression mutants to detection of BafA passenger domain in the culture supernatant. **b**, Detection of Strep-tagged protein from the eluted fractions of Strep-Tactin affinity chromatography by immunoblotting. An arrowhead indicted the position of reactive band. Similar results were obtained in 3 independent experiments. **c**, Schematic of predicted domains and amino acid sequence of BafA. Green highlights show the peptides identified using nano-LC-MS/MS in the eluted fraction #3 of Strep-Tactin affinity chromatography. See also Supplementary Table 1. Source data are provided as a Source Data file.

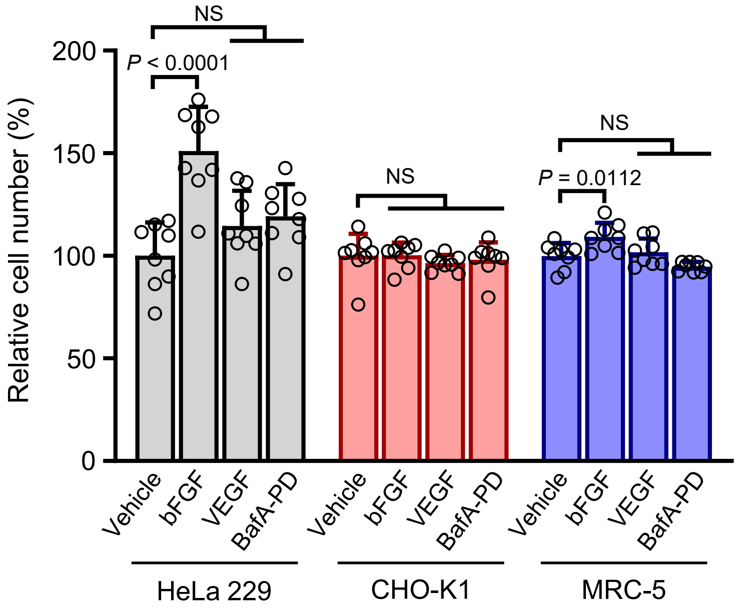

Supplementary Figure 6. Cell specificity of mitogenic activity of BafA. HeLa 229 (human cervical epithelial cells), CHO-K1 (Chinese hamster ovary cells) and MRC-5 (human lung fibroblasts) were treated with PBS (vehicle), bFGF (20 ng/mL), VEGF (20 ng/mL) or BafA-PD (100 ng/mL). Three days after treatment, the cell numbers were counted. Bars are colored based on the cell lines: grey for HeLa 229, red for CHO-K1 and blue for MRC-5. Data are mean ± s.d. (n = 8 biological replicates). Statistical significance was determined using one-way ANOVA with Dunnett's multiple comparisons test. NS, not significant. Source data are provided as a Source Data file.

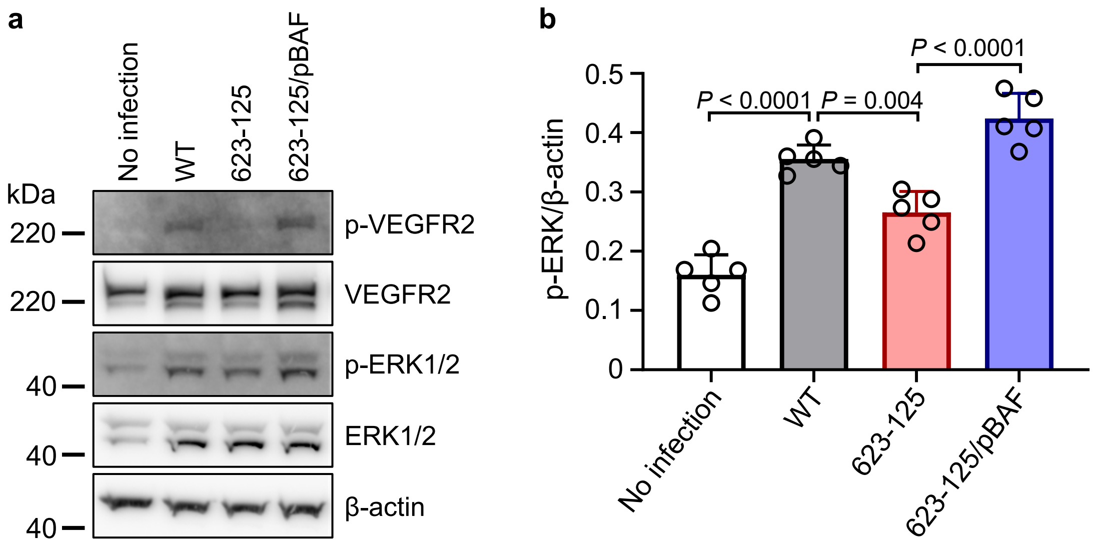

Supplementary Figure 7. Production of BafA from *Bh* induces phosphorylation of VEGFR2 and increases the level of phosphorylated ERK1/2 in HUVECs. a, HUVECs were infected with *Bh* (WT, 623-125, or 623-125/pBAF) at MOI of 500 for 24 h. The cells were harvested, lysed, and then subjected to immunoblotting using antibody against p-VEGFR2, VEGFR2, p-ERK1/2, ERK1/2, or β-actin. Similar results were obtained in 5 independent experiments. **b,** Quantification of p-ERK1/2 represented as the ratio of p-ERK1/2 to β-actin. Bars are coloured based on the strains: white for no infection, grey for WT, red for 623-125 and blue for 623-125/pBAF. Data are mean ± s.d. (n = 5 biological replicates). Statistical significance was determined using one-way ANOVA with Tukey's multiple comparisons test. Source data are provided as a Source Data file.

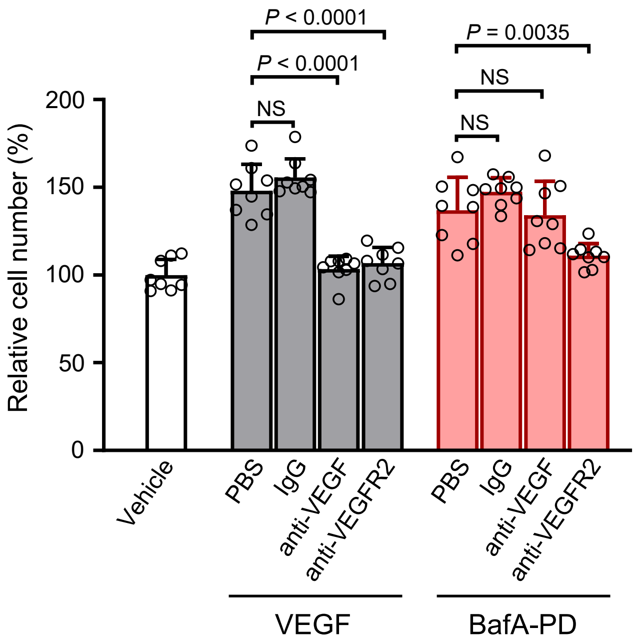

Supplementary Figure 8. The anti-VEGFR2 antibody inhibits mitogenic activity of BafA. HUVECs were treated with VEGF (20 ng/mL) or BafA-PD (50 ng/mL) in the presence of PBS, normal human IgG (3 μg/mL), anti-VEGF antibody (Bevacizumab, 3 μg/mL), or anti-VEGFR2 antibody (Ramucirumab, 3 μg/mL). Forty-eight hours after treatment, the cell numbers were counted. Bars are coloured based on the treatment: white for vehicle, grey for VEGF and blue for BafA-PD. Data are mean ± s.d. (n = 8 biological replicates). Statistical significance was determined using one-way ANOVA with Dunnett's multiple comparisons test. NS, not significant. Source data are provided as a Source Data file.

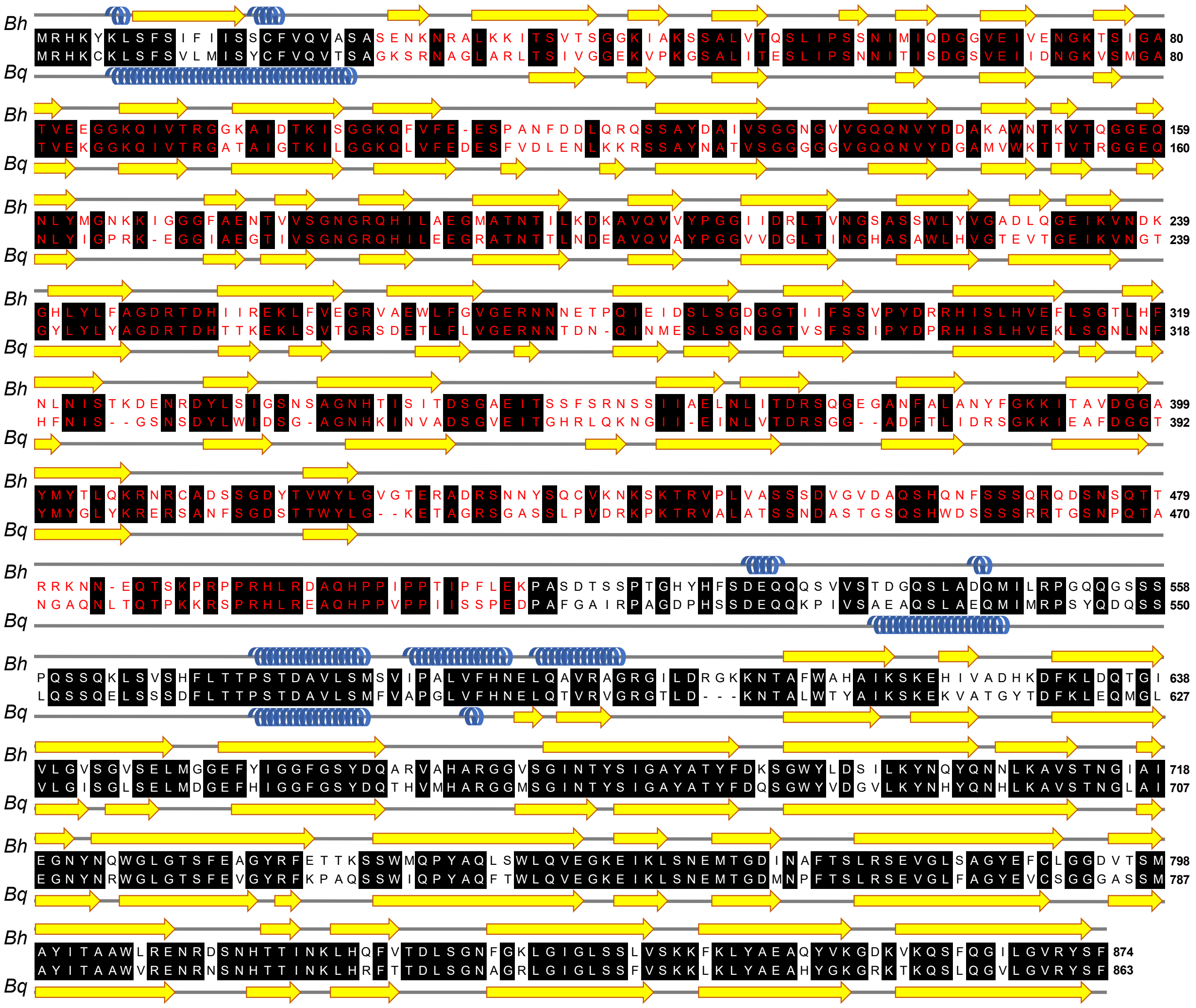

Supplementary Figure 9. Sequence alignment of BafA autotransporter derived from *Bh* and *Bq*. Full length sequences of *B. henselae* (*Bh*)- and *B. quintana* (*Bq*)-derived BafA autotransporters are aligned. Letters highlighted in black indicate matched amino acid residues between the two sequences. The red letters indicate the region expressed as recombinant proteins, which had amino acid identity of 59.7% between the two proteins. The blue spirals (α-helix) and yellow arrows (β-strand) represent the secondary structure predicted by PSIPRED v4.0 on the PSIPRED server (http://bioinf.cs.ucl.ac.uk/psipred/).

Percentage of identity

Min – Max

		1	2	3	4	5	6	7	8	9	10	11	12	13	14	15	16	17	18	19	20	21	22	23	24	25	26	27	28	29	30	31	32	33
B. tribocorum	1		69.57	66.19	68.62	68.38	64.12	65.19	61.46	58.66	54.50	51.30	50.26	53.66	51.13	54.74	53.30	38.61	37.09	37.25	33.80	35.23	24.31	24.75	23.56	23.43	24.92	7.06	8.53	4.88	4.17	3.48	2.11	2.81
B. rattimassiliensis	2	0.36		67.38	68.03	65.90	63.94	62.88	59.96	57.73	56.28	50.09	50.79	50.17	51.30	52.72	53.66	37.22	36.92	35.40	32.41	34.02	23.66	23.80	22.83	22.42	24.13	6.16	8.59	4.23	3.34	3.70	2.13	2.48
B. taylorii	3	0.41	0.39		65.96	65.24	61.50	63.52	60.21	55.75	57.77	53.73	51.66	53.83	53.39	55.44	54.86	38.09	36.40	36.53	31.11	34.16	23.33	23.62	22.16	21.48	24.72	7.10	8.55	4.73	3.67	3.85	2.47	3.35
B. massiliensis	4	0.36	0.36	0.40		68.38	65.19	63.94	62.34	56.36	54.14	49.83	47.90	51.05	49.13	54.93	51.22	35.53	34.89	34.17	31.86	34.61	22.63	23.07	22.32	21.22	23.97	6.83	8.47	3.89	2.65	2.30	1.78	2.14
B. grahamii	5	0.38	0.40	0.43	0.36		73.75	66.43	60.39	56.54	54.67	50.87	50.35	50.09	50.00	52.90	52.35	36.63	35.12	36.30	30.83	32.55	23.59	23.01	22.27	21.74	23.78	7.26	8.06	3.85	3.85	3.33	1.94	3.00
B. elizabethae	6	0.44	0.43	0.49	0.41	0.31		71.48	58.26	53.00	52.56	49.13	48.78	51.31	50.70	51.49	51.30	36.28	35.99	36.13	31.25	34.31	22.29	21.85	22.27	21.48	25.67	6.94	8.55	4.73	4.02	4.55	2.47	2.65
B. mastomydis	7	0.43	0.45	0.45	0.43	0.41	0.34		58.97	56.01	54.32	50.52	50.00	50.96	51.92	53.43	51.83	36.63	35.47	37.08	31.07	32.80	23.73	24.31	22.84	22.65	24.69	7.25	7.42	4.37	4.01	3.85	2.29	2.99
B. florencae	8	0.49	0.50	0.50	0.46	0.50	0.54	0.53		58.41	55.83	51.56	50.35	51.13	54.28	55.91	52.79	37.67	37.54	36.58	32.83	35.09	22.61	23.62	21.73	21.58	24.65	6.43	8.21	4.54	3.48	3.32	1.94	2.64
B. birtlesii	9	0.51	0.52	0.56	0.54	0.54	0.61	0.55	0.51		53.67	49.83	48.25	48.78	50.00	49.74	50.78	33.91	35.17	35.01	30.61	32.94	22.25	22.69	23.11	21.17	24.32	7.32	8.93	4.60	3.89	3.91	2.33	2.86
B. vinsonii	10	0.56	0.52	0.50	0.55	0.55	0.59	0.56	0.54	0.59		51.22	49.30	52.44	53.48	53.25	53.55	35.58	35.80	36.79	32.18	33.19	23.65	23.21	23.67	22.87	25.00	6.46	6.98	4.70	4.33	3.44	2.01	3.10
B. henselae	11	0.64	0.66	0.59	0.66	0.65	0.69	0.66	0.64	0.66	0.61		65.38	62.09	58.96	57.74	50.86	36.14	37.56	37.40	31.28	34.20	22.92	22.78	21.59	20.93	26.67	6.08	7.38	4.29	4.29	3.26	2.08	3.45
B. koehlerae	12	0.67	0.65	0.64	0.72	0.67	0.71	0.68	0.67	0.70	0.66	0.42		58.74	59.69	55.23	50.69	37.48	39.07	38.86	31.32	33.53	22.99	22.70	20.68	20.61	26.13	6.25	8.04	4.14	3.61	2.76	2.09	2.60
B. senegalensis	13	0.61	0.67	0.60	0.65	0.68	0.65	0.66	0.66	0.69	0.59	0.47	0.53		59.69	55.75	52.34	38.10	38.49	36.96	31.73	33.82	24.36	23.50	22.05	21.51	26.05	6.88	8.35	4.48	4.12	3.96	2.26	2.77
B. quintana	14	0.65	0.63	0.59	0.68	0.67	0.65	0.63	0.59	0.66	0.56	0.51	0.50	0.51		57.42	53.46	37.02	37.59	37.63	31.04	32.75	23.28	22.99	21.97	20.64	22.45	6.75	8.35	4.65	3.78	3.80	2.09	3.14
B. washoeensis	15	0.58	0.61	0.56	0.57	0.61	0.64	0.60	0.56	0.66	0.57	0.52	0.56	0.56	0.52		52.25	38.93	39.07	37.79	33.56	35.87	24.16	24.75	23.81	21.48	25.71	5.64	7.09	4.04	2.98	2.81	1.95	2.30
B. doshiae	16	0.61	0.60	0.57	0.64	0.62	0.65	0.64	0.62	0.64	0.56	0.66	0.67	0.64	0.60	0.61		36.43	36.47	36.33	32.88	34.97	23.43	24.00	21.81	20.45	23.99	5.42	7.97	4.30	3.77	3.95	1.91	2.94
B. melophagi	17	0.87	0.90	0.88	0.94	0.92	0.93	0.92	0.89	0.98	0.90	0.94	0.90	0.88	0.91	0.84	0.93		67.78	60.88	30.71	31.77	27.25	27.69	24.10	23.62	26.57	4.70	7.20	3.85	3.66	3.68	2.04	3.15
B. schoenbuchensis	18	0.93	0.92	0.94	0.97	0.98	0.95	0.97	0.91	0.95	0.91	0.91	0.87	0.88	0.91	0.85	0.94	0.37		60.14	30.71	32.94	26.76	27.06	24.51	22.48	27.43	4.30	6.98	3.60	3.24	3.07	2.74	2.73
B. bovis	19	0.90	0.95	0.93	0.98	0.93	0.94	0.91	0.92	0.95	0.87	0.91	0.87	0.92	0.89	0.87	0.93	0.42	0.43		30.36	32.10	28.47	26.91	23.94	21.97	27.40	3.95	5.83	3.44	2.75	2.94	1.74	2.78
B. clarridgeiae	20	0.83	0.86	0.92	0.88	0.93	0.92	0.92	0.86	0.92	0.83	0.91	0.90	0.89	0.91	0.83	0.85	0.87	0.89	0.89		53.20	22.17	22.53	19.95	19.00	20.96	5.10	5.74	2.79	3.06	3.21	1.83	2.39
B. rochalimae	21	0.81	0.85	0.85	0.84	0.90	0.84	0.89	0.81	0.87	0.83	0.84	0.86	0.85	0.88	0.79	0.81	0.86	0.84	0.86	0.53		22.58	22.45	20.83	19.93	23.46	4.37	6.95	2.85	2.85	2.70	2.27	2.87
B. queenslandensis	22	0.95	0.98	0.99	1.04	0.98	1.04	0.97	1.03	1.06	0.95	1.01	1.01	0.94	0.99	0.97	0.98	0.84	0.89	0.74	0.90	0.93		69.86	38.78	34.86	21.83	3.81	5.69	4.04	3.67	3.68	1.79	2.50
B. alsatica	23	0.93	0.97	0.98	1.02	1.00	1.06	0.95	0.98	1.03	0.97	1.02	1.02	0.98	1.00	0.94	0.95	0.82	0.87	0.79	0.88	0.93	0.35		39.20	34.76	22.83	3.17	5.06	3.87	3.85	3.34	1.80	2.51
B. australis	24	0.94	0.98	1.00	1.01	0.99	1.00	0.97	1.03	0.97	0.96	1.03	1.08	1.00	1.01	0.93	1.01	0.95	0.95	0.89	0.99	1.00	0.87	0.86		33.39	21.09	4.55	6.13	4.98	4.43	4.28	2.55	3.45
B. ancashensis	25	0.90	0.94	1.00	1.02	0.99	1.00	0.94	0.99	1.03	0.96	1.02	1.04	0.99	1.04	1.00	1.05	0.91	0.98	0.93	0.99	0.97	0.89	0.89	0.99		20.35	3.86	5.29	3.51	3.66	3.04	2.44	2.11
B. bacilliformis	26	1.01	1.05	1.03	1.07	1.07	0.99	1.03	1.03	1.05	0.98	0.93	0.96	0.96	1.10	0.99	1.06	0.93	0.90	0.89	1.02	0.93	1.10	1.05	1.13	1.14		4.52	4.96	3.50	3.11	2.95	2.57	2.57
VEGF-D	27	2.14	2.35	2.14	2.21	2.11	2.17	2.11	2.27	2.09	2.19	2.39	2.34	2.20	2.23	2.47	2.56	2.85	3.02	3.09	2.47	2.92	3.08	3.54	2.59	2.73	2.69		34.80	6.34	7.11	5.98	5.42	6.25
VEGF-C	28	2.17	2.16	2.16	2.19	2.24	2.16	2.37	2.21	2.09	2.41	2.41	2.27	2.22	2.20	2.43	2.26	2.45	2.51	2.70	2.56	2.40	2.68	2.89	2.47	2.56	2.87	0.85		6.42	6.88	4.35	4.40	5.12
VEGF-B	29	2.20	2.44	2.26	2.59	2.58	2.26	2.37	2.30	2.30	2.31	2.37	2.44	2.32	2.23	2.51	2.38	2.63	2.72	2.73	2.77	2.84	2.45	2.52	2.13	2.50	2.91	1.95	2.16		30.77	27.78	20.75	20.57
VEGF-A	30	2.63	3.09	2.88	3.76	2.79	2.71	2.71	2.97	2.77	2.63	2.57	2.88	2.64	2.76	3.36	2.80	2.95	3.18	3.51	2.81	3.08	2.81	2.72	2.48	2.62	3.49	1.87	2.24	1.09		30.13	25.11	21.28
PlGF	31	2.88	2.79	2.70	4.16	2.99	2.43	2.70	2.98	2.70	2.99	3.00	3.40	2.63	2.69	3.40	2.63	2.87	3.23	3.24	2.64	3.10	2.72	2.92	2.49	2.91	3.62	2.05	2.93	1.14	1.14		21.97	21.52
VEGF-F	32	3.10	3.10	2.78	3.60	3.31	2.80	2.93	3.29	2.93	3.31	3.10	3.10	2.94	3.10	3.31	3.33	3.29	2.64	3.56	2.96	2.64	3.58	3.58	2.76	2.63	2.93	2.11	2.21	1.22	0.92	1.13		28.21
VEGF-E	33	2.37	2.59	2.12	2.89	2.28	2.49	2.28	2.46	2.37	2.28	2.05	2.48	2.39	2.20	2.72	2.29	2.27	2.46	2.37	2.32	2.12	2.59	2.59	2.10	2.69	2.72	1.80	1.89	1.17	0.99	1.04	1.07	

Distance

Min – Max

Supplementary Figure 10. Diversity of amino acid sequences between passenger domains of BafA-homologous proteins and VEGF family. The upper comparison shows the percentage of overlapping alignment positions where the two sequences agree, and the lower comparison exhibits the Jukes-Cantor corrected distance between the two sequences. These BafA-homologous proteins were selected from top hits of a Protein BLAST search using BafA-PD as the query sequence. See also Supplementary Table 4. The red letters indicate the species used in this study. Source data are provided as a Source Data file.

Supplementary Table 1. List of all peptides identified using nano-LC-MS/MS in the eluate of Strap-Tactin affinity chromatography

Accession	Description	Replicate 1		Replicate 2		Total		
		# Peptides	# PSMs[a]	# Peptides	# PSMs	# Peptides	# PSMs	Coverage (%)
A0A0H3LXX9[b]	Uncharacterized protein	15	40	14	38	17	78	22.4
A0A0H3M489	Expressed protein	4	7	2	4	4	11	27.2
A0A0H3LXV5	Uncharacterized protein	3	5	1	2	3	7	26.3
Q6G2F0	Glycine cleavage system H protein	1	2	2	6	3	8	23.0
A0A0R4J7J8	Uncharacterized protein	2	4	0	0	2	4	12.3
A0A0H3LW86	Phenylalanine-specific permease	0	0	1	1	1	1	2.3
A0A0H3M3N0	Amino-acid ABC transporter binding protein	1	2	1	2	1	4	5.0
Q6G1T4	TrwJ-like protein	1	1	0	0	1	1	3.1
A0A0H3LYC3	Biotin carboxyl carrier protein of acetyl-CoA carboxylase	1	1	0	0	1	1	4.3
A0A0H3LXE4	NADH-quinone oxidoreductase	1	1	0	0	1	1	1.3
A0A0R4J8M0	Uncharacterized protein	1	1	0	0	1	1	3.4

[a]PSM, peptide spectral matches, filtered at a 1% false discovery rate.

[b]A0A0H3LL9 corresponds to BafA referred in this study.

Supplementary Table 2. List of BadA- and T4SS-related peptides detected in whole cell lysate of *B. henselae* Houson-1 by nano-LC-MS/MS

Accession	Gene	Description	Replicate 1			Replicate 2			Replicate 3		
			# Peptides	# PSMs[a]	Coverage (%)	# Peptides	# PSMs	Coverage (%)	# Peptides	# PSMs	Coverage (%)
A0A0H3LVF3	BH01490 (badA1)	Surface protein/Bartonella adhesin	31	32	28.7	31	37	28.1	32	33	28.1
A0A0H3LX70	BH01510 (badA1)	Surface protein/Bartonella adhesin	10	11	8.3	17	20	16.3	7	7	5.1
Q9R2W4	BH13280 (virB4)	Type IV secretion system protein virB4	0	0	0	1	1	2.3	0	0	0
A0A0H3LXV5	BH13380 (virD4)	Type IV secretion system-coupling protein VirD4	0	0	0	0	0	0	1	1	1.5

[a]PSM, peptide spectral matches, filtered at a 1% false discovery rate.

Supplementary Table 3. Primers used in this study

Primer	Sequence (5'–3')	Application
mariner-F	GCCGCAGTCGGTAACCAGTTCTGCTAACAGGTTGGATGATAAGT	pMariK
mariner-R	ATGCGGTACCTCTAGACCGCGCCACATAACAGGTTGGATGATAAG	
rpsL-F	GGCCGCAGTCGGTAACCATTAATTCCCGGTTTGACTGGTC	
TPase-R	AGCAGAACTGGTTACCCGTTCAGGCTGCGCAACTGTTGG	
TpR-F	TAATATTTGCCCATGGCACGAACCCAGTTGACATAAGC	pBAF, pSBAF
TpR-R	CAGGGGATCAAGATCTCAGCGGGCGGCGAAGCC	
RS02720-Prom-Fw	TAGAACTAGTGGATCGTTTATACGTTTATTTTCTGGTCAAT	
pBBR-RS02720-Rv	GCCTTAGCTCCTGATATCAAAAACTATAGCGTACCCCTAAAATAC	
strep-bafA-Fw	TGGAGCCACCCGCAGTTCGAAAAACTGCAGAGTGAGAATAAGAATAGGGCG	
bafA-SP-Rv	CTGCGGGTGGCTCCAACTCGCGCTTGCAACTTGCAC	
NheI-Bh-Fw	TCTCGCTAGCAGTGAGAATAAGAATAGGGCG	pET-28b-BH513
SalI-Bh513-Rv	GACTGTCGACTCATTTTTCTAAAAATGGAATCGTC	
NheI-Bq-Fw	TCTCGCTAGCGGTAAAAGTAGGAATGCAGGGCTTGCAAGACTGACAAGTATA G	pET-28b-BQ505
SalI-Bq505-Rv	GACTGTCGACTCAATCTTCTGGAGAGGAGATAATGGG	
Mari1	TCAATTCGAGCTCGGGTATCGC	Inverse-PCR
Mari4	ACTCGTCCAACATCAATACAACC	

Supplementary Table 4. List of BafA homologues and the members of VEGF family

Organism	Description	Accession
Bartonella henselae	Autotransporter outer membrane beta-barrel domain-containing protein	WP_011180481
Bartonella koehlerae	Autotransporter outer membrane beta-barrel domain-containing protein	WP_034458933
Bartonella senegalensis	Autotransporter, partial	WP_034988522
Bartonella washoeensis	Autotransporter outer membrane beta-barrel domain-containing protein	WP_006923152
Bartonella quintana	Autotransporter outer membrane beta-barrel domain-containing protein	WP_034450980
Bartonella taylorii	Autotransporter outer membrane beta-barrel domain-containing protein	WP_078692231
Bartonella florencae	hypothetical protein, partial	WP_019219656
Bartonella tribocorum	Autotransporter outer membrane beta-barrel domain-containing protein	WP_100130036
Bartonella vinsonii	Autotransporter outer membrane beta-barrel domain-containing protein	WP_015398914
Bartonella rattimassiliensis	Autotransporter outer membrane beta-barrel domain-containing protein	WP_026088004
Bartonella mastomydis	Autotransporter outer membrane beta-barrel domain-containing protein	WP_139413051
Bartonella massiliensis	Autotransporter outer membrane beta-barrel domain-containing protein	WP_142416305
Bartonella grahamii	Autotransporter outer membrane beta-barrel domain-containing protein	WP_114648068
Bartonella birtlesii	Autotransporter outer membrane beta-barrel domain-containing protein	WP_006589304
Bartonella elizabethae	Autotransporter outer membrane beta-barrel domain-containing protein	WP_005773720
Bartonella doshiae	Autotransporter outer membrane beta-barrel domain-containing protein	WP_074380818
Bartonella rochalimae	Autotransporter outer membrane beta-barrel domain-containing protein	WP_035007285
Bartonella clarridgeiae	Autotransporter outer membrane beta-barrel domain-containing protein	WP_013544909
Bartonella bovis	Autotransporter outer membrane beta-barrel domain-containing protein	WP_010701038
Bartonella schoenbuchensis	Autotransporter outer membrane beta-barrel domain-containing protein	WP_010703578
Bartonella melophagi	Autotransporter outer membrane beta-barrel domain-containing protein	WP_007477514
Bartonella bacilliformis	Autotransporter outer membrane beta-barrel domain-containing protein	WP_005766622
Bartonella queenslandensis	Autotransporter outer membrane beta-barrel domain-containing protein	WP_039759906
Bartonella ancashensis	Putative autotransporter	ALE03961
Bartonella alsatica	Autotransporter outer membrane beta-barrel domain-containing protein	WP_083832384
Bartonella australis	Autotransporter outer membrane beta-barrel domain-containing protein	WP_015397864
Homo sapiens	Vascular endothelial growth factor A (VEGF-A)	P15692
Homo sapiens	Vascular endothelial growth factor B (VEGF-B)	P49765
Homo sapiens	Vascular endothelial growth factor C (VEGF-C)	CAA63907
Homo sapiens	Vascular endothelial growth factor D (VEGF-D)	O43915
Orf virus	Vascular endothelial growth factor E (VEGF-E)	ABA00650
Bothrops insularis	Snake venom vascular endothelial growth factor toxin (VEGF-F)	Q90X24
Homo sapiens	Placenta growth factor (PlGF)	P049763
