# Supplementary material for: The Bartonella autotransporter BafA activates the host VEGF pathway to drive angiogenesis
Source: Nat Commun. 2020 Jul 16;11:3571. doi: 10.1038/s41467-020-17391-2 (PMC7366657; doi:10.1038/s41467-020-17391-2)
Supplement: Supplementary file 3 — Reporting Summary [file 41467_2020_17391_MOESM3_ESM.pdf]

nature research

Corresponding author(s): Kentaro Tsukamoto, and Yohei Doi

Last updated by author(s): Jun 21, 2020

Reporting Summary

Nature Research wishes to improve the reproducibility of the work that we publish. This form provides structure for consistency and transparency in reporting. For further information on Nature Research policies, see Authors & Referees and the Editorial Policy Checklist .

Statistics

For all statistical analyses, confirm that the following items are present in the figure legend, table legend, main text, or Methods section.

n/a	Confirmed	
☐	☒	The exact sample size (*n*) for each experimental group/condition, given as a discrete number and unit of measurement
☐	☒	A statement on whether measurements were taken from distinct samples or whether the same sample was measured repeatedly
☐	☒	The statistical test(s) used AND whether they are one- or two-sided<br>*Only common tests should be described solely by name; describe more complex techniques in the Methods section.*
☒	☐	A description of all covariates tested
☒	☐	A description of any assumptions or corrections, such as tests of normality and adjustment for multiple comparisons
☐	☒	A full description of the statistical parameters including central tendency (e.g. means) or other basic estimates (e.g. regression coefficient) AND variation (e.g. standard deviation) or associated estimates of uncertainty (e.g. confidence intervals)
☐	☒	For null hypothesis testing, the test statistic (e.g. *F*, *t*, *r*) with confidence intervals, effect sizes, degrees of freedom and *P* value noted<br>*Give P values as exact values whenever suitable.*
☒	☐	For Bayesian analysis, information on the choice of priors and Markov chain Monte Carlo settings
☒	☐	For hierarchical and complex designs, identification of the appropriate level for tests and full reporting of outcomes
☒	☐	Estimates of effect sizes (e.g. Cohen's *d*, Pearson's *r*), indicating how they were calculated

Our web collection on statistics for biologists contains articles on many of the points above.

Software and code

Policy information about availability of computer code

Data collection	Harmony 4.5 was used to collect fluorescence images.
Data analysis	GraphPad Prism 8 was used to analyze the data and generate graphs. Harmony 4.5 was used to analyze fluorescence images. SignalP 4.0 server and NCBI's Conserved Domain Database were used to analyse amino acid sequences. The bcl2fastq 1.8.4 software was used for base-calling. CLC Genomic Workbench 8.5.2 was used for quality trimming of raw RNA-seq reads, alignment of trimmed reads to the human reference genome, and statistical analysis of DEGs. DAVID 6.8 was used for GO analysis. Heatmapper (http://heatmapper.ca/) was used to generate the heatmaps. The fast5 output of Nanopore sequencing was base-called using Guppy base caller and de-multiplexed using Guppy barcoder. Unicycler v. 0.4.8 was used for de novo assembly of B. henselae genomes. Multi Gauge ver. 3.0 was used to calculate the density of the reactive bands in immunoblots. CLC Main Workbench 8.1.3 was used to perform the pairwise comparisons of BafA-homologous proteins and to make phylogenetic tree. The peptide ions were detected using MS in the data-dependent acquisition mode with the Xcalibur software (version 4.0; Thermo Scientific). The MS/MS searches were performed using MASCOT (Version 2.6.1, Matrix Science) and SEQUEST HT search algorithms against the SwissProt and TrEMBL Bartonella henselae protein databases (v2017-10-25) using Proteome Discoverer 2.2 (Ver. 2.2.0.388; Thermo Scientific). PSIPRED v4.0 on the PSIPRED server (http://bioinf.cs.ucl.ac.uk/psipred/) was used to predict the secondary structure.

For manuscripts utilizing custom algorithms or software that are central to the research but not yet described in published literature, software must be made available to editors/reviewers. We strongly encourage code deposition in a community repository (e.g. GitHub). See the Nature Research guidelines for submitting code & software for further information.

Data

Policy information about availability of data

All manuscripts must include a data availability statement. This statement should provide the following information, where applicable:

- Accession codes, unique identifiers, or web links for publicly available datasets
- A list of figures that have associated raw data
- A description of any restrictions on data availability

The RNA-seq raw data for each sample reported in this work have been deposited in the DDBJ Sequence Read Archive with the accession no. DRA009444 (https://ddbj.nig.ac.jp/DRASearch/submission?acc=DRA009444). The draft genome sequences of B. henselae transposants 623-125 and 804-29 can be found in the DDBJ with the accession no. BLJS01000000 (http://getentry.ddbj.nig.ac.jp/getentry/na/BLJS01000000/?format=flatfile&filetype=html&trace=true&show_suppressed=false&limit=10) and BLJT01000000 (http://getentry.ddbj.nig.ac.jp/getentry/na/BLJT01000000/?format=flatfile&filetype=html&trace=true&show_suppressed=false&limit=10), respectively. The MS raw datasets for the culture supernatant and the whole cell extracts have been deposited in the ProteomeXchange Consortium64 via the jPOST65 partner repository under data-set identifiers PXD017507 (http://proteomecentral.proteomexchange.org/cgi/GetDataset?ID=PXD017507) and PXD018354 (http://proteomecentral.proteomexchange.org/cgi/GetDataset?ID=PXD018354), respectively. The source data underlying Figs 1a, c, e, g, 2a–f, 3b-k, 4a-k and 5b-e and Supplementary Figs 2, 3, 4a-b, 5b, 6, 7a-b, 8 and 10 are provided as a Source Data file.

Field-specific reporting

Please select the one below that is the best fit for your research. If you are not sure, read the appropriate sections before making your selection.

[x] Life sciences [ ] Behavioural & social sciences [ ] Ecological, evolutionary & environmental sciences

For a reference copy of the document with all sections, see nature.com/documents/nr-reporting-summary-flat.pdf

Life sciences study design

All studies must disclose on these points even when the disclosure is negative.

Sample size	No formal statistical methods were used to predetermine sample size. Sample sizes were determined based on preliminary data, our previous experience and similar studies of other groups, and sufficient to carry out the experiments required for statistical analysis. In addition, each figure legend describes the number of and biological replicates (between n=3 and n=8 independent samples for each group).
Data exclusions	No data were excluded from the analyses.
Replication	The experiments were repeated at least 3 times for all analyses. Number of reproductions of the experimental finding is in figure legends particularly important wherever results from representative experiments (such as micrographs) are shown. All attempts at replication were successful.
Randomization	Allocation of mice to different groups was random.
Blinding	Blinding was not relevant for cell-based assay because there was no group allocation. Investigators were not blinded to allocation during mouse assay, because it was not possible to be blinded the mice due to the obvious color differences on the skin of the Matrigel transplant region between control and experimental groups.

Reporting for specific materials, systems and methods

We require information from authors about some types of materials, experimental systems and methods used in many studies. Here, indicate whether each material, system or method listed is relevant to your study. If you are not sure if a list item applies to your research, read the appropriate section before selecting a response.

Materials & experimental systems

n/a	Involved in the study	
[ ]	[x]	Antibodies
[ ]	[x]	Eukaryotic cell lines
[x]	[ ]	Palaeontology
[ ]	[x]	Animals and other organisms
[x]	[ ]	Human research participants
[x]	[ ]	Clinical data

Methods

n/a	Involved in the study	
[x]	[ ]	ChIP-seq
[x]	[ ]	Flow cytometry
[x]	[ ]	MRI-based neuroimaging

Antibodies

Antibodies used	Rabbit anti-BafA polyclonal antibody was generated in this study. Rat anti-mouse CD31 (clone# MEC13.3, BD Pharmingen, catalog# 553370, lot# 8043575 1:500), rabbit anti-VEGFR2 (55B11, Cell Signalling Technology, 2479, lot# 18, 1:1,000), rabbit anti-phospho-VEGFR2 (Tyr1175) (19A10, Cell Signalling Technology, 2478, lot# 15, 1:1,000), rabbit anti-MEK1/2 (D1A5, Cell Signalling Technology, 8277, lot# 5, 1:1,000), rabbit anti-phospho-MEK1/2 (Ser217/221) (41G9, Cell Signalling Technology, 9154, lot# 18, 1:1,000), rabbit anti-ERK1/2 (137F5, Cell Signalling Technology, 4695, lot# 21, 1:1,000), rabbit anti-phospho-ERK1/2 (Thr202/Tyr204) (D13.14.4E, Cell Signalling Technology, 4370, lot# 17, 1:2,000), rabbit anti-Akt (pan) (C67E7, Cell Signalling Technology, 4691, lot# 20, 1:1,000), rabbit anti-phospho-Akt (Ser473) (D9E, Cell Signalling Technology, 4060, lot# 23, 1:1,000), β-actin (13E5, Cell Signalling Technology, 4970, lot# 15, 1:1,000), Purified mouse anti-Strep-tag II monoclonal antibody (MBL, #M211-3, lot# 002, at), human anti-VEGF (BEVACIZUMAB BS Intravenous Infusion 100mg [Pfizer], Pfizer, at 3 μg/mL), and human anti-VEGFR2 (Cyramza Injection, Eli Lilly Japan, at 3 μg/mL). HRP-conjugated donkey anti-rabbit IgG (Jackson ImmunoResearch, 711-035-152, lot# 130173, 1:4,000), HRP-conjugated goat anti-mouse IgG (Jackson ImmunoResearch, 115-035-062, lot# 123311 1:4,000), Alexa488-conjugated goat anti-rat IgG (Invitrogen, A11006, lot# 2048174, 1:500).
Validation	All commercial antibodies were validated by the respective suppliers. The validation statements are available on the manufacturers' websites.<br>Anti-CD31 (species: mouse; application: FC, IP, IHC); anti-VEGFR2 (species: human, mouse; application: WB, IP, IHC, IF); anti-phospho-VEGFR2 (species: human, mouse; application: WB, IHC, IF); anti-MEK1/2 (species: human, mouse, rat, etc.; application: WB, IF, FC); anti-phospho-MEK1/2 (species: human, mouse, rat, mokey; application: WB, IP); anti-ERK1/2 (species: human, mouse, rat, etc.; application: WB, IP, IHC, IF, FC); anti-phospho-ERK1/2 (species: human, mouse, rat, etc.; application: WB, IP, IHC, IF, FC); anti-Akt (species: human, mouse, rat, etc.; application: WB, IP, IHC, IF, FC); anti-phospho-Akt (species: human, mouse, rat, etc.; application: WB, IP, IHC, IF, FC); and β-actin (species: human, mouse, rat, etc.; application: WB, IHC, IF, FC). Neutralizing activities of human anti-VEGF (Bevacizumab) and human anti-VEGFR2 (Cyramza) are sufficiently validated, since they are both approved for medical use to treat a number of cancer.<br>For anti-BafA antibody, the antibody were first tested by WB and confirmed the reactive band toward purified recombinant BafA-PD.

Eukaryotic cell lines

Policy information about cell lines

Cell line source(s)	HUVEC (PromoCell: C-12203); HeLa 229 (JCRB cell bank: JCRB9086); CHO-K1 (JCRB cell bank: IFO50414); MRC-5 (JCRB cell bank: JCRB9008).
Authentication	All cell lines were authenticated by the suppliers.
Mycoplasma contamination	All cell lines were tested negative for mycoplasma contamination by the suppliers.
Commonly misidentified lines (See ICLAC register)	No commonly misidentified cell lines were used in this study.

Animals and other organisms

Policy information about studies involving animals; ARRIVE guidelines recommended for reporting animal research

Laboratory animals	8-week-old female C57BL/6J mice were purchased from Japan SLC. Mice were housed under 12 light/12 dark cycle, ambient temperatures of 22 ± 2°C with 55 ± 10% humidity. A Japanese White rabbit (strain JW; 10-week-old female; Kitayama Labes Co., Ltd.) was immunized by EveBioscience Ltd (Japan).
Wild animals	The study did not involve wild animals.
Field-collected samples	The study did not involve samples collected from the field.
Ethics oversight	All animal experiments were approved by the Institutional Animal Care and Use Committee of the Fujita Health University, and carried out according to the Regulations for the Management of Laboratory Animals at Fujita Health University.

Note that full information on the approval of the study protocol must also be provided in the manuscript.
